# Crystal structure of bis­{(*Z*)-(benzyl­amino)[(5*Z*)-2-(benzyl­imino-κ*N*)-5-(2-meth­oxy-2-oxo­ethyl­idene)-4-oxo­thio­lan-3-yl­idene]methane­thiol­ato-κ*S*}copper(II)

**DOI:** 10.1107/S2056989015005022

**Published:** 2015-03-21

**Authors:** Konstantin Obydennov, Liliya Khamidullina, Pavel Slepukhin, Yury Morzherin

**Affiliations:** a Ural Federal University, Mira 19 Ekaterinburg 620002, Russian Federation; b I. Postovsky Institute of Organic Synthesis, Kovalevskoy 22 Ekaterinburg 620090, Russian Federation

**Keywords:** crystal structure, copper(II) complex, thio­amide

## Abstract

The title complex, [Cu(C_22_H_19_N_2_O_3_S_2_)_2_], was obtained from the reaction between (*Z*)-methyl 2-(5-benzyl­imino-4-benzyl­carbamo­thioyl-3-oxo­thio­lan-2-yl­idene)acetate and Cu(NO_3_)_2_. The Cu^II^ atom is tetra­coordinated by two *N*,*S*-bidentate ligands, forming a highly distorted tetra­hedral environment. The structure displays two intra­molecular N—H⋯O hydrogen bonds.

## Related literature   

For synthesis and applications of thio­amide complexes, see: Jiang *et al.* (2013 ▸); Zieliński & Jurczak (2005 ▸); Arena *et al.* (2001 ▸); Shamkhy *et al.* (2013 ▸). For the importance of copper in biological systems, see: Siegel (1973 ▸); Mohan *et al.* (1998 ▸). For the synthesis of the title compound, see: Obydennov *et al.* (2013 ▸).
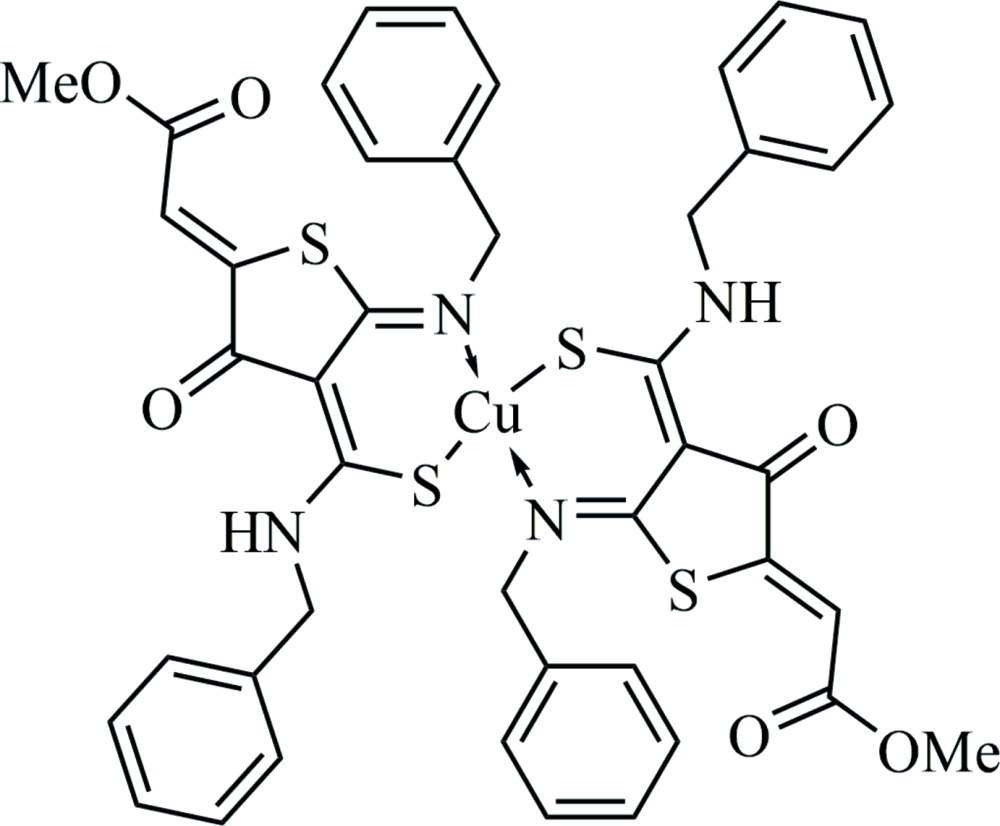


## Experimental   

### Crystal data  

[Cu(C_22_H_19_N_2_O_3_S_2_)_2_]
*M* *_r_* = 910.56
Triclinic, 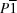

*a* = 10.7595 (6) Å
*b* = 11.6318 (5) Å
*c* = 18.8162 (8) Å
α = 104.846 (4)°
β = 91.038 (4)°
γ = 109.119 (4)°
*V* = 2137.25 (18) Å^3^
*Z* = 2
Mo *K*α radiation
μ = 0.76 mm^−1^
*T* = 295 K
0.28 × 0.11 × 0.03 mm

### Data collection  

Agilent Xcalibur Eos diffractometer
Absorption correction: multi-scan (*SADABS*; Sheldrick, 1996 ▸) *T*_min_ = 0.769, *T*_max_ = 1.000
20682 measured reflections
11592 independent reflections
5788 reflections with *I* > 2σ(*I*)
*R*_int_ = 0.032

### Refinement  

*R*[*F* ^2^ > 2σ(*F* ^2^)] = 0.058
*wR*(*F* ^2^) = 0.162
*S* = 1.01
11592 reflections
534 parameters
18 restraints
H-atom parameters constrained
Δρ_max_ = 0.55 e Å^−3^
Δρ_min_ = −0.26 e Å^−3^

### 

Data collection: *CrysAlis PRO* (Agilent, 2011 ▸); cell refinement: *CrysAlis PRO*; data reduction: *CrysAlis PRO*; program(s) used to solve structure: *SUPERFLIP* (Palatinus & Chapuis, 2007 ▸); program(s) used to refine structure: *SHELXS97* (Sheldrick, 2008 ▸); molecular graphics: *OLEX2* (Dolomanov *et al.*, 2009 ▸); software used to prepare material for publication: *OLEX2* (Dolomanov *et al.*, 2009 ▸) and *publCIF* (Westrip, 2010 ▸).

## Supplementary Material

Crystal structure: contains datablock(s) I, exp_221. DOI: 10.1107/S2056989015005022/lr2133sup1.cif

Structure factors: contains datablock(s) I. DOI: 10.1107/S2056989015005022/lr2133Isup2.hkl

**Fig. 1.**
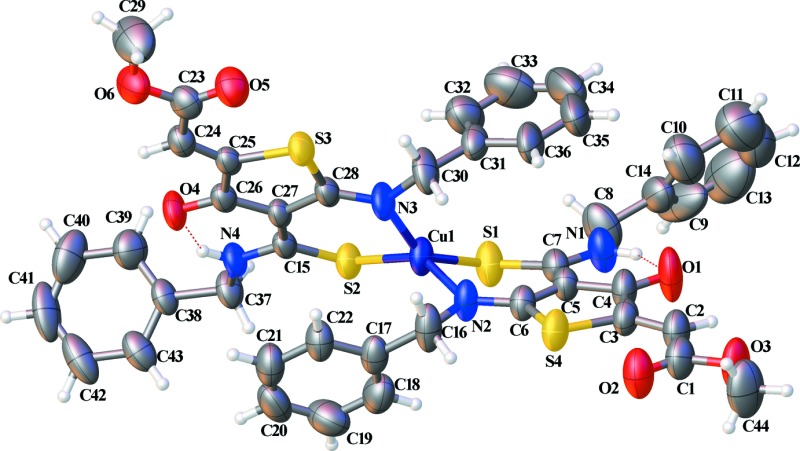
The molecular structure of (I), with atom labels and 50% probability displacement ellipsoids for non-H atoms.

. DOI: 10.1107/S2056989015005022/lr2133fig1.tif
The mol­ecular structure of (I), with atom labels and 50% probability displacement ellipsoids for non-H atoms.

CCDC reference: 1039799

Additional supporting information:  crystallographic information; 3D view; checkCIF report

## Figures and Tables

**Table:** 

Cu1S1	2.2387(10)
Cu1S2	2.2341(8)
Cu1N2	1.976(3)
Cu1N3	1.969(3)

**Table:** 

S2Cu1S1	91.69(3)
N2Cu1S1	96.02(8)
N2Cu1S2	144.94(9)
N3Cu1S1	144.11(9)
N3Cu1S2	97.17(7)
N3Cu1N2	96.32(12)

**Table 2:** Hydrogen-bond geometry (, )

*D*H*A*	*D*H	H*A*	*D* *A*	*D*H*A*
N1H1O1	0.86	1.87	2.604(4)	142
N4H4O4	0.86	1.88	2.612(3)	141

## supplementary crystallographic information

### S1. Structural commentary

The structure of the title complex, (I), C_44_H_38_CuN_4_O_6_S_4_, has triclinic (*P*1) symmetry. The ligands form two six-membered cycles with the including in the coordination of the N-atoms of the benzyl­amino-groups and S-atoms of the thio­amide moiety (Fig.1). Metallocycles are non-planar, been in the "pseudo-cover" conformation in which Cu-atoms are deviated from the least-squared planes of the atoms S1N2C6C5C7 and C15C23C27N3S2 on the distances 0.697 and 0.548 Å accordingly. The nearest coordination of the central ion is the distorted tetra­hedron (Table 2). Deviations from the ideal in the tetra­hedron geometry are very serious, so the coordination of the Cu^2+^-ion is may regard also as distorted squared. In this case S-atoms with inter­atomic angle S1Cu1S2 91.7^o^ occupy the cis-positions toward Cu-atom. Due to strong π-conjugation in the metallocycle, the bonds lengths of the 2p-atoms in the metallocycles have the medium magnitude between the thio­enamine and mercaptoenimine configurations, and we can`t make conclusion about dominated form of the ligand in the complex. Most principal elements of the structure which together the central ion ordered the conformation of the ligands are intra­molecular H-bond NH···O between NH-group of the thio­amide moiety and CO-group in the thio­phene moiety, and also polar S···O contact between the S-atom in the thio­phene ring and CO-group of the COOMe-substituent which. This contact fixed COOMe-substituent in the plane of the thio­phene moietiy. No any shortened inter­molecular contacts in the crystal presented.

### S2. Synthesis and crystallization

The title complex was synthesized by the addition of Cu(NO_3_)_2_ (1 mmol) to an ethanol - chloro­form solution of (Z)-methyl 2-(5-(benzyl­amino)-4-(benzyl­carbamo­thioyl)-3-oxothien-2(3H)-yl­idene)acetate (2 mmol) (Obydennov *et al.*, 2013). The mixture was stirred for 5 hr. The resulting deep green solution was filtered and left to evaporate at room temperature. The crystalline complex, which deposited upon standing for several days, and dark green prismatic crystals were isolated. M.p. 175-177 °C.

### S3. Refinement

The non-hydrogen atoms were refined in the anisotropic approximation, hydrogen atoms were included in the refinement isotropically in the "riding" model with C—H = 0.93 Å for aryl, 0.96 Å for methine and 0.96 Å for methyl H atoms, respectively. U_iso_(H) = 1.2Ueq(C) for aryl and methine, and 1.5Ueq(C) for methyl H atoms. Final results of the refinement: R_1_ = 0.0577, wR_2_ = 0.1280 [I>=2σ (I)], R_1_ = 0.1278, wR_2_ = 0.1623 (all data), GooF= 1.005, Δρ_ē_= 0.55/-0.26 ē/Å^3^.

### Crystal data

**Table:**

[Cu(C_22_H_19_N_2_O_3_S_2_)_2_]	*F*(000) = 942
*M**_r_* = 910.56	*D*_x_ = 1.415 Mg m^−^^3^
Triclinic, *P*1	Melting point = 448–450 K
*a* = 10.7595 (6) Å	Mo *K*α radiation, λ = 0.7107 Å
*b* = 11.6318 (5) Å	Cell parameters from 4392 reflections
*c* = 18.8162 (8) Å	θ = 2.2–28.9°
α = 104.846 (4)°	µ = 0.76 mm^−^^1^
β = 91.038 (4)°	*T* = 295 K
γ = 109.119 (4)°	Plate, brown–green
*V* = 2137.25 (18) Å^3^	0.28 × 0.11 × 0.03 mm
*Z* = 2	

### Data collection

**Table:**

Agilent Xcalibur Eos diffractometer	11592 independent reflections
Radiation source: Enhance (Mo) X-ray Source	5788 reflections with *I* > 2σ(*I*)
Graphite monochromator	*R*_int_ = 0.032
Detector resolution: 15.9555 pixels mm^-1^	θ_max_ = 30.8°, θ_min_ = 1.9°
ω scans	*h* = −9→15
Absorption correction: multi-scan (*SADABS*; Sheldrick, 1996)	*k* = −16→16
*T*_min_ = 0.769, *T*_max_ = 1.000	*l* = −26→26
20682 measured reflections	

### Refinement

**Table:**

Refinement on *F*^2^	Primary atom site location: iterative
Least-squares matrix: full	Secondary atom site location: difference Fourier map
*R*[*F*^2^ > 2σ(*F*^2^)] = 0.058	Hydrogen site location: inferred from neighbouring sites
*wR*(*F*^2^) = 0.162	H-atom parameters constrained
*S* = 1.00	*w* = 1/[σ^2^(*F*_o_^2^) + (0.0657*P*)^2^ + 0.020*P*] where *P* = (*F*_o_^2^ + 2*F*_c_^2^)/3
11592 reflections	(Δ/σ)_max_ = 0.001
534 parameters	Δρ_max_ = 0.55 e Å^−^^3^
18 restraints	Δρ_min_ = −0.26 e Å^−^^3^

### Special details

**Table:**

Geometry. All e.s.d.'s (except the e.s.d. in the dihedral angle between two l.s. planes) are estimated using the full covariance matrix. The cell e.s.d.'s are taken into account individually in the estimation of e.s.d.'s in distances, angles and torsion angles; correlations between e.s.d.'s in cell parameters are only used when they are defined by crystal symmetry. An approximate (isotropic) treatment of cell e.s.d.'s is used for estimating e.s.d.'s involving l.s. planes.
Refinement. Refinement of *F*^2^ against ALL reflections. The weighted *R*-factor *wR* and goodness of fit *S* are based on *F*^2^, conventional *R*-factors *R* are based on *F*, with *F* set to zero for negative *F*^2^. The threshold expression of *F*^2^ > σ(*F*^2^) is used only for calculating *R*-factors(gt) *etc*. and is not relevant to the choice of reflections for refinement. *R*-factors based on *F*^2^ are statistically about twice as large as those based on *F*, and *R*-factors based on ALL data will be even larger.

### Fractional atomic coordinates and isotropic or equivalent isotropic displacement parameters (Å^2^)

**Table:**

	*x*	*y*	*z*	*U*_iso_*/*U*_eq_	
Cu1	0.40787 (4)	0.74099 (3)	0.72941 (2)	0.05996 (16)	
S1	0.51282 (11)	0.79437 (8)	0.84313 (6)	0.0751 (3)	
S2	0.38829 (9)	0.53810 (6)	0.71147 (5)	0.0575 (2)	
S3	0.40369 (9)	0.68404 (6)	0.47870 (5)	0.0562 (2)	
S4	0.27743 (11)	1.08801 (7)	0.80003 (5)	0.0688 (3)	
O1	0.5766 (3)	1.2189 (2)	0.94766 (17)	0.1086 (11)	
O2	0.1979 (3)	1.3090 (2)	0.83944 (17)	0.0977 (10)	
O3	0.3119 (3)	1.4830 (2)	0.92986 (17)	0.1020 (10)	
O4	0.2511 (3)	0.32945 (17)	0.46259 (12)	0.0670 (7)	
O5	0.3904 (3)	0.6636 (2)	0.32537 (14)	0.0771 (8)	
O6	0.3091 (3)	0.4783 (2)	0.23764 (14)	0.0789 (8)	
N1	0.6358 (3)	1.0149 (3)	0.93368 (19)	0.0833 (10)	
H1	0.6444	1.0925	0.9547	0.100*	
N2	0.3141 (3)	0.8641 (2)	0.75264 (15)	0.0634 (8)	
N3	0.4323 (3)	0.7478 (2)	0.62691 (15)	0.0619 (8)	
N4	0.2820 (3)	0.33696 (19)	0.60191 (15)	0.0540 (7)	
H4	0.2546	0.2986	0.5560	0.065*	
C1	0.2875 (5)	1.3650 (3)	0.8881 (2)	0.0803 (12)	
C11	1.0348 (8)	1.2765 (7)	1.0241 (5)	0.167 (3)	
H11	1.0853	1.3488	1.0116	0.201*	
C2	0.3841 (4)	1.3137 (3)	0.9089 (2)	0.0759 (11)	
H2	0.4481	1.3637	0.9485	0.091*	
C3	0.3868 (4)	1.1983 (3)	0.87429 (19)	0.0652 (10)	
C4	0.4870 (4)	1.1502 (3)	0.8979 (2)	0.0709 (11)	
C5	0.4632 (3)	1.0215 (3)	0.85765 (18)	0.0579 (9)	
C6	0.3552 (4)	0.9737 (3)	0.80132 (18)	0.0560 (8)	
C7	0.5387 (4)	0.9518 (3)	0.87919 (19)	0.0616 (9)	
C8	0.7285 (4)	0.9667 (4)	0.9613 (3)	0.0999 (15)	
H8A	0.7535	0.9121	0.9202	0.120*	
H8B	0.6867	0.9165	0.9939	0.120*	
C9	0.8502 (4)	1.0727 (4)	1.0027 (2)	0.0849 (12)	
C12	1.0621 (7)	1.2424 (7)	1.0856 (5)	0.146 (3)	
H12	1.1302	1.3058	1.1188	0.176*	
C14	0.9028 (6)	1.0608 (7)	1.0646 (3)	0.143 (3)	
H14	0.8572	0.9890	1.0791	0.172*	
C15	0.3333 (3)	0.4624 (2)	0.62032 (17)	0.0461 (7)	
C13	1.0132 (8)	1.1404 (9)	1.1077 (4)	0.172 (3)	
H13	1.0502	1.1253	1.1479	0.206*	
C10	0.9071 (6)	1.1758 (5)	0.9789 (4)	0.140 (2)	
H10	0.8706	1.1858	0.9367	0.169*	
C16	0.1912 (4)	0.8263 (3)	0.7030 (2)	0.0810 (13)	
H16A	0.2130	0.8451	0.6564	0.097*	
H16B	0.1367	0.8746	0.7253	0.097*	
C17	0.1150 (4)	0.6869 (3)	0.6886 (2)	0.0626 (9)	
C18	0.0831 (4)	0.6326 (4)	0.7450 (2)	0.0837 (12)	
H18	0.1079	0.6826	0.7937	0.100*	
C19	0.0137 (5)	0.5034 (5)	0.7308 (3)	0.0981 (14)	
H19	−0.0076	0.4675	0.7698	0.118*	
C20	−0.0225 (4)	0.4300 (4)	0.6599 (3)	0.0880 (13)	
H20	−0.0679	0.3435	0.6501	0.106*	
C21	0.0076 (4)	0.4832 (3)	0.6037 (2)	0.0821 (12)	
H21	−0.0180	0.4329	0.5552	0.099*	
C22	0.0758 (4)	0.6110 (3)	0.6174 (2)	0.0696 (10)	
H22	0.0955	0.6462	0.5780	0.083*	
C23	0.3386 (3)	0.5502 (3)	0.3077 (2)	0.0616 (9)	
C24	0.3035 (3)	0.4749 (3)	0.35970 (18)	0.0539 (8)	
H24	0.2595	0.3881	0.3413	0.065*	
C25	0.3315 (3)	0.5246 (2)	0.43259 (18)	0.0468 (7)	
C26	0.3026 (3)	0.4466 (2)	0.48604 (17)	0.0491 (8)	
C27	0.3422 (3)	0.5206 (2)	0.56072 (16)	0.0440 (7)	
C28	0.3958 (3)	0.6539 (2)	0.56731 (18)	0.0496 (8)	
C29	0.3578 (6)	0.5428 (5)	0.1827 (3)	0.124 (2)	
H29A	0.3697	0.4837	0.1397	0.185*	
H29B	0.4410	0.6090	0.2023	0.185*	
H29C	0.2952	0.5787	0.1695	0.185*	
C44	0.2253 (6)	1.5477 (4)	0.9119 (3)	0.123 (2)	
H44A	0.2314	1.5521	0.8617	0.185*	
H44B	0.2519	1.6319	0.9447	0.185*	
H44C	0.1356	1.5018	0.9174	0.185*	
C30	0.4953 (4)	0.8788 (3)	0.6213 (2)	0.0805 (13)	
H30A	0.4336	0.9242	0.6326	0.097*	
H30B	0.5149	0.8756	0.5708	0.097*	
C31	0.6208 (4)	0.9498 (3)	0.67281 (19)	0.0654 (10)	
C32	0.7061 (5)	0.8926 (4)	0.6841 (3)	0.0973 (14)	
H32	0.6863	0.8067	0.6613	0.117*	
C33	0.8238 (6)	0.9612 (7)	0.7295 (4)	0.132 (2)	
H33	0.8830	0.9213	0.7369	0.159*	
C34	0.8531 (7)	1.0867 (7)	0.7634 (4)	0.143 (3)	
H34	0.9319	1.1326	0.7941	0.171*	
C35	0.7679 (7)	1.1433 (5)	0.7522 (3)	0.127 (2)	
H35	0.7879	1.2291	0.7752	0.152*	
C36	0.6503 (5)	1.0765 (3)	0.7069 (2)	0.0904 (14)	
H36	0.5915	1.1170	0.6995	0.108*	
C37	0.2678 (4)	0.2582 (2)	0.65237 (19)	0.0633 (10)	
H37A	0.2112	0.2787	0.6895	0.076*	
H37B	0.3537	0.2731	0.6773	0.076*	
C38	0.2073 (4)	0.1210 (2)	0.60763 (19)	0.0555 (9)	
C39	0.2769 (5)	0.0647 (3)	0.5591 (2)	0.0826 (12)	
H39	0.3645	0.1094	0.5552	0.099*	
C40	0.2169 (7)	−0.0599 (4)	0.5151 (3)	0.1066 (18)	
H40	0.2631	−0.0979	0.4810	0.128*	
C41	0.0899 (7)	−0.1246 (4)	0.5230 (3)	0.108 (2)	
H41	0.0495	−0.2074	0.4939	0.129*	
C42	0.0218 (5)	−0.0716 (4)	0.5718 (3)	0.0971 (16)	
H42	−0.0643	−0.1179	0.5773	0.117*	
C43	0.0803 (4)	0.0518 (3)	0.6137 (2)	0.0744 (11)	
H43	0.0322	0.0889	0.6470	0.089*	

### Atomic displacement parameters (Å^2^)

**Table:**

	*U*^11^	*U*^22^	*U*^33^	*U*^12^	*U*^13^	*U*^23^
Cu1	0.0769 (3)	0.03305 (19)	0.0597 (3)	0.01695 (19)	−0.0049 (2)	−0.00118 (16)
S1	0.0958 (8)	0.0461 (4)	0.0716 (6)	0.0212 (5)	−0.0190 (5)	0.0024 (4)
S2	0.0704 (6)	0.0339 (4)	0.0607 (5)	0.0152 (4)	0.0009 (4)	0.0046 (3)
S3	0.0655 (6)	0.0324 (3)	0.0623 (5)	0.0100 (3)	0.0039 (4)	0.0079 (3)
S4	0.0893 (8)	0.0414 (4)	0.0684 (6)	0.0259 (4)	−0.0060 (5)	−0.0005 (4)
O1	0.110 (3)	0.0588 (15)	0.116 (2)	0.0235 (15)	−0.044 (2)	−0.0350 (15)
O2	0.112 (3)	0.0607 (15)	0.101 (2)	0.0280 (16)	−0.0212 (19)	−0.0062 (15)
O3	0.126 (3)	0.0490 (13)	0.114 (2)	0.0398 (16)	−0.0192 (19)	−0.0171 (14)
O4	0.0852 (18)	0.0291 (10)	0.0644 (15)	0.0005 (10)	−0.0023 (12)	0.0010 (9)
O5	0.090 (2)	0.0573 (14)	0.0767 (18)	0.0144 (13)	0.0028 (14)	0.0200 (12)
O6	0.0743 (19)	0.0867 (17)	0.0542 (16)	0.0073 (14)	−0.0073 (13)	0.0105 (13)
N1	0.079 (2)	0.0547 (16)	0.094 (3)	0.0169 (16)	−0.0216 (19)	−0.0076 (16)
N2	0.076 (2)	0.0408 (13)	0.0623 (18)	0.0227 (13)	−0.0179 (15)	−0.0069 (12)
N3	0.083 (2)	0.0261 (11)	0.0617 (17)	0.0068 (12)	−0.0001 (15)	0.0037 (11)
N4	0.0609 (18)	0.0276 (11)	0.0609 (17)	0.0046 (11)	0.0007 (13)	0.0054 (10)
C1	0.099 (4)	0.0474 (19)	0.083 (3)	0.023 (2)	0.002 (3)	0.0006 (19)
C11	0.151 (6)	0.125 (5)	0.201 (7)	0.028 (4)	0.042 (5)	0.027 (5)
C2	0.091 (3)	0.0440 (18)	0.077 (3)	0.0186 (19)	−0.003 (2)	−0.0031 (16)
C3	0.081 (3)	0.0397 (16)	0.062 (2)	0.0153 (17)	0.0063 (19)	−0.0011 (14)
C4	0.083 (3)	0.0433 (17)	0.068 (2)	0.0154 (18)	−0.006 (2)	−0.0075 (16)
C5	0.059 (2)	0.0421 (16)	0.059 (2)	0.0117 (15)	0.0011 (17)	−0.0008 (14)
C6	0.069 (2)	0.0382 (15)	0.054 (2)	0.0169 (15)	0.0003 (17)	0.0045 (13)
C7	0.062 (2)	0.0500 (17)	0.057 (2)	0.0094 (16)	0.0015 (17)	0.0006 (15)
C8	0.078 (3)	0.083 (3)	0.126 (4)	0.019 (2)	−0.031 (3)	0.022 (3)
C9	0.061 (3)	0.093 (3)	0.074 (3)	0.014 (2)	−0.003 (2)	−0.004 (2)
C12	0.123 (5)	0.113 (4)	0.153 (6)	0.017 (4)	0.019 (4)	−0.018 (4)
C14	0.085 (4)	0.257 (8)	0.058 (3)	0.053 (4)	−0.004 (3)	0.003 (4)
C15	0.0424 (18)	0.0281 (13)	0.0605 (19)	0.0082 (12)	0.0069 (14)	0.0048 (12)
C13	0.153 (6)	0.226 (7)	0.114 (5)	0.077 (5)	−0.017 (4)	−0.003 (5)
C10	0.116 (5)	0.095 (4)	0.175 (6)	0.017 (4)	0.004 (4)	0.004 (4)
C16	0.095 (3)	0.0490 (18)	0.085 (3)	0.0317 (19)	−0.029 (2)	−0.0108 (17)
C17	0.061 (2)	0.0516 (18)	0.069 (2)	0.0272 (16)	−0.0134 (18)	−0.0030 (16)
C18	0.082 (3)	0.084 (3)	0.068 (3)	0.026 (2)	−0.003 (2)	−0.005 (2)
C19	0.075 (3)	0.104 (4)	0.102 (4)	0.008 (3)	0.013 (3)	0.036 (3)
C20	0.057 (3)	0.062 (2)	0.122 (4)	0.0059 (19)	−0.004 (3)	0.005 (3)
C21	0.086 (3)	0.062 (2)	0.078 (3)	0.021 (2)	−0.013 (2)	−0.008 (2)
C22	0.078 (3)	0.0512 (19)	0.067 (2)	0.0195 (18)	−0.013 (2)	0.0004 (16)
C23	0.051 (2)	0.062 (2)	0.066 (2)	0.0148 (17)	−0.0035 (17)	0.0150 (17)
C24	0.050 (2)	0.0418 (15)	0.062 (2)	0.0105 (14)	0.0005 (16)	0.0084 (14)
C25	0.0395 (18)	0.0347 (14)	0.061 (2)	0.0113 (12)	0.0046 (15)	0.0066 (13)
C26	0.0425 (19)	0.0360 (14)	0.063 (2)	0.0111 (13)	0.0051 (15)	0.0063 (13)
C27	0.0422 (18)	0.0273 (12)	0.0555 (18)	0.0083 (12)	0.0035 (14)	0.0043 (12)
C28	0.046 (2)	0.0313 (13)	0.062 (2)	0.0078 (13)	0.0047 (15)	0.0050 (13)
C29	0.139 (5)	0.128 (4)	0.070 (3)	0.000 (4)	0.002 (3)	0.031 (3)
C44	0.148 (5)	0.069 (3)	0.150 (5)	0.059 (3)	−0.024 (4)	−0.003 (3)
C30	0.117 (4)	0.0311 (15)	0.073 (3)	0.0042 (18)	−0.011 (2)	0.0094 (15)
C31	0.086 (3)	0.0375 (16)	0.060 (2)	0.0082 (17)	0.0143 (19)	0.0073 (14)
C32	0.084 (4)	0.069 (3)	0.128 (4)	0.019 (3)	0.017 (3)	0.019 (3)
C33	0.079 (4)	0.151 (5)	0.165 (6)	0.032 (4)	0.014 (4)	0.050 (5)
C34	0.089 (5)	0.151 (6)	0.113 (5)	−0.026 (4)	0.009 (4)	−0.004 (4)
C35	0.119 (5)	0.069 (3)	0.125 (5)	−0.022 (3)	0.027 (4)	−0.019 (3)
C36	0.108 (4)	0.0363 (18)	0.096 (3)	0.001 (2)	0.015 (3)	−0.0032 (18)
C37	0.077 (3)	0.0331 (15)	0.068 (2)	0.0060 (15)	−0.0014 (18)	0.0117 (14)
C38	0.063 (2)	0.0306 (14)	0.064 (2)	0.0051 (15)	−0.0056 (17)	0.0134 (14)
C39	0.095 (3)	0.053 (2)	0.097 (3)	0.024 (2)	0.015 (3)	0.017 (2)
C40	0.181 (6)	0.061 (3)	0.087 (3)	0.061 (3)	0.009 (3)	0.011 (2)
C41	0.169 (6)	0.031 (2)	0.097 (4)	0.007 (3)	−0.041 (4)	0.012 (2)
C42	0.097 (4)	0.055 (2)	0.113 (4)	−0.013 (2)	−0.037 (3)	0.034 (2)
C43	0.071 (3)	0.0506 (19)	0.091 (3)	0.0036 (18)	−0.009 (2)	0.0266 (19)

### Geometric parameters (Å, º)

**Table:**

Cu1—S1	2.2387 (10)	C5—C7	1.439 (5)
Cu1—S2	2.2341 (8)	C8—C9	1.502 (5)
Cu1—N2	1.976 (3)	C9—C14	1.344 (7)
Cu1—N3	1.969 (3)	C9—C10	1.348 (7)
S1—C7	1.707 (3)	C12—C13	1.308 (10)
S2—C15	1.707 (3)	C14—C13	1.336 (8)
S3—C25	1.732 (3)	C15—C27	1.441 (4)
S3—C28	1.788 (3)	C16—C17	1.508 (5)
S4—C3	1.737 (4)	C17—C18	1.363 (5)
S4—C6	1.792 (3)	C17—C22	1.372 (5)
O1—C4	1.234 (4)	C18—C19	1.393 (6)
O2—C1	1.201 (5)	C19—C20	1.356 (6)
O3—C1	1.332 (4)	C20—C21	1.350 (6)
O3—C44	1.459 (5)	C21—C22	1.377 (5)
O4—C26	1.244 (3)	C23—C24	1.450 (5)
O5—C23	1.203 (4)	C24—C25	1.331 (4)
O6—C23	1.335 (4)	C25—C26	1.492 (4)
O6—C29	1.437 (5)	C26—C27	1.419 (4)
N1—C7	1.325 (4)	C27—C28	1.437 (4)
N1—C8	1.441 (5)	C30—C31	1.502 (5)
N2—C6	1.295 (4)	C31—C32	1.340 (6)
N2—C16	1.476 (4)	C31—C36	1.373 (4)
N3—C28	1.297 (4)	C32—C33	1.385 (7)
N3—C30	1.484 (4)	C33—C34	1.361 (8)
N4—C15	1.326 (3)	C34—C35	1.334 (8)
N4—C37	1.457 (4)	C35—C36	1.382 (8)
C1—C2	1.449 (6)	C37—C38	1.514 (4)
C11—C12	1.371 (9)	C38—C39	1.365 (5)
C11—C10	1.541 (9)	C38—C43	1.364 (5)
C2—C3	1.343 (4)	C39—C40	1.399 (6)
C3—C4	1.475 (5)	C40—C41	1.358 (8)
C4—C5	1.429 (4)	C41—C42	1.338 (7)
C5—C6	1.419 (5)	C42—C43	1.374 (5)
			
S2—Cu1—S1	91.69 (3)	N4—C15—C27	116.5 (3)
N2—Cu1—S1	96.02 (8)	C27—C15—S2	126.54 (19)
N2—Cu1—S2	144.94 (9)	C12—C13—C14	110.7 (8)
N3—Cu1—S1	144.11 (9)	C9—C10—C11	117.7 (7)
N3—Cu1—S2	97.17 (7)	N2—C16—C17	110.8 (3)
N3—Cu1—N2	96.32 (12)	C18—C17—C16	121.7 (3)
C7—S1—Cu1	105.69 (13)	C18—C17—C22	118.3 (3)
C15—S2—Cu1	107.41 (10)	C22—C17—C16	120.1 (4)
C25—S3—C28	92.49 (14)	C17—C18—C19	120.9 (4)
C3—S4—C6	91.48 (16)	C20—C19—C18	119.8 (4)
C1—O3—C44	116.2 (3)	C21—C20—C19	119.7 (4)
C23—O6—C29	115.6 (3)	C20—C21—C22	120.9 (4)
C7—N1—C8	126.5 (3)	C17—C22—C21	120.5 (4)
C6—N2—Cu1	126.7 (2)	O5—C23—O6	123.9 (3)
C6—N2—C16	119.7 (3)	O5—C23—C24	124.1 (3)
C16—N2—Cu1	113.34 (19)	O6—C23—C24	111.9 (3)
C28—N3—Cu1	127.4 (2)	C25—C24—C23	122.9 (3)
C28—N3—C30	119.7 (3)	C24—C25—S3	126.2 (2)
C30—N3—Cu1	112.9 (2)	C24—C25—C26	123.0 (3)
C15—N4—C37	126.0 (3)	C26—C25—S3	110.8 (2)
O2—C1—O3	124.4 (4)	O4—C26—C25	119.7 (3)
O2—C1—C2	124.7 (3)	O4—C26—C27	127.6 (3)
O3—C1—C2	110.9 (4)	C27—C26—C25	112.8 (2)
C12—C11—C10	109.2 (7)	C26—C27—C15	121.4 (2)
C3—C2—C1	123.9 (4)	C26—C27—C28	112.3 (3)
C2—C3—S4	126.4 (3)	C28—C27—C15	126.3 (3)
C2—C3—C4	121.8 (3)	N3—C28—S3	119.8 (2)
C4—C3—S4	111.8 (2)	N3—C28—C27	128.6 (3)
O1—C4—C3	120.3 (3)	C27—C28—S3	111.6 (2)
O1—C4—C5	127.8 (4)	N3—C30—C31	112.4 (3)
C5—C4—C3	111.9 (3)	C32—C31—C30	121.1 (3)
C4—C5—C7	120.4 (3)	C32—C31—C36	119.7 (4)
C6—C5—C4	112.6 (3)	C36—C31—C30	119.2 (4)
C6—C5—C7	126.8 (3)	C31—C32—C33	120.1 (5)
N2—C6—S4	120.3 (3)	C34—C33—C32	120.4 (6)
N2—C6—C5	127.7 (3)	C35—C34—C33	119.4 (6)
C5—C6—S4	112.0 (2)	C34—C35—C36	121.1 (5)
N1—C7—S1	116.6 (3)	C31—C36—C35	119.4 (5)
N1—C7—C5	117.0 (3)	N4—C37—C38	108.1 (3)
C5—C7—S1	126.4 (3)	C39—C38—C37	121.0 (3)
N1—C8—C9	111.1 (4)	C43—C38—C37	120.4 (3)
C14—C9—C8	117.5 (5)	C43—C38—C39	118.6 (3)
C14—C9—C10	120.4 (5)	C38—C39—C40	120.2 (4)
C10—C9—C8	122.0 (5)	C41—C40—C39	118.9 (5)
C13—C12—C11	134.0 (8)	C42—C41—C40	121.5 (4)
C13—C14—C9	127.4 (8)	C41—C42—C43	119.4 (5)
N4—C15—S2	116.9 (2)	C38—C43—C42	121.4 (4)

### Hydrogen-bond geometry (Å, º)

**Table:**

*D*—H···*A*	*D*—H	H···*A*	*D*···*A*	*D*—H···*A*
N1—H1···O1	0.86	1.87	2.604 (4)	142
N4—H4···O4	0.86	1.88	2.612 (3)	141
