# Supplementary figures and images for: Crystal structure of bis­{(Z)-(benzyl­amino)[(5Z)-2-(benzyl­imino-κN)-5-(2-meth­oxy-2-oxo­ethyl­idene)-4-oxo­thio­lan-3-yl­idene]methane­thiol­ato-κS}copper(II)
Source: Acta Crystallogr E Crystallogr Commun. 2015 Mar 21;71(Pt 4):m93–4. doi: 10.1107/S2056989015005022 (PMC4438821; doi:10.1107/S2056989015005022)

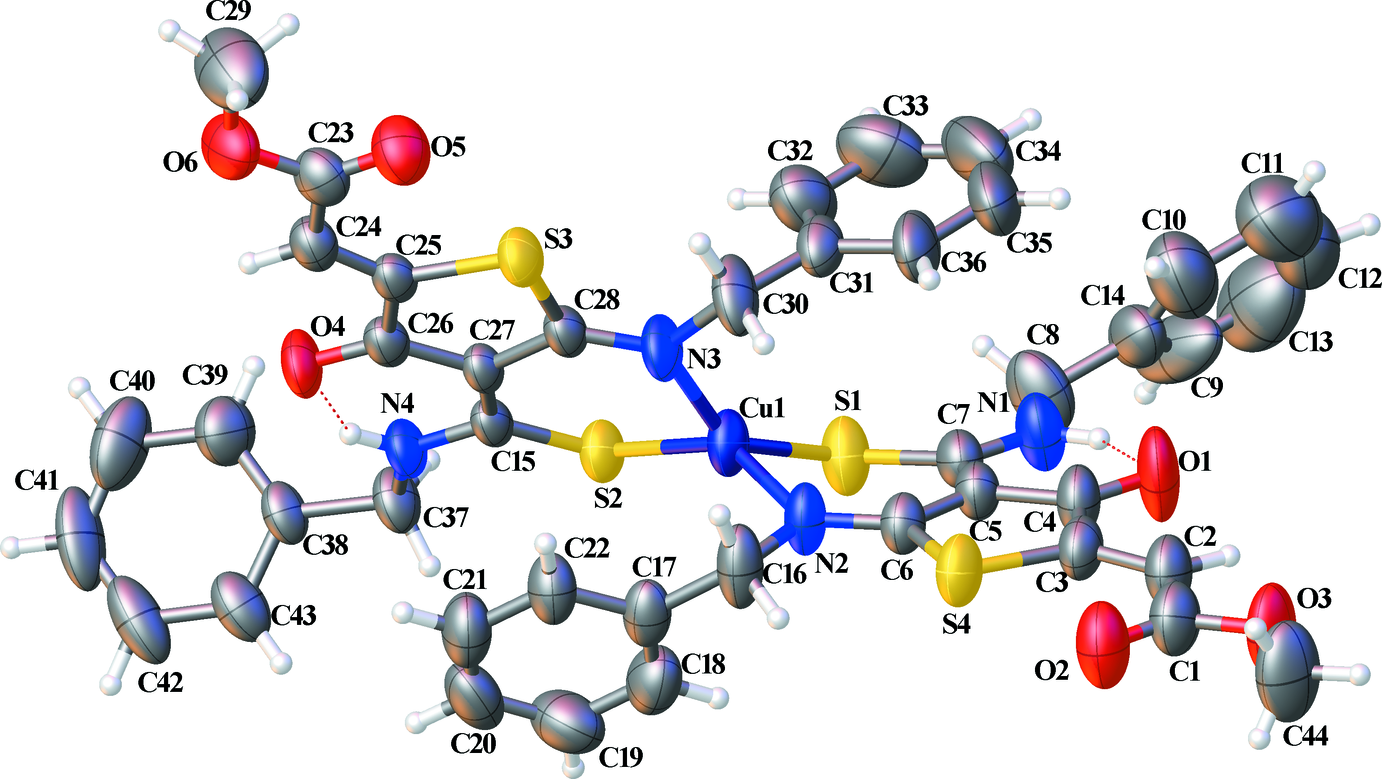

Supplement: Supplementary file 3 [file e-71-00m93-fig1.tif]
